# C-terminal short arginine/serine repeat sequence-dependent regulation of Y14 (RBM8A) localization

**DOI:** 10.1038/s41598-017-18765-1

**Published:** 2018-01-12

**Authors:** Takanori Tatsuno, Yasuhito Ishigaki

**Affiliations:** Medical Research Institute, Kanazawa Medical University, Kahoku, Ishikawa, 920-0293 Japan

## Abstract

Y14 (RBM8A) is an RNA recognition motif-containing protein that forms heterodimers with MAGOH and serves as a core factor of the RNA surveillance machinery for the exon junction complex (EJC). The role of the Y14 C-terminal serine/arginine (RS) repeat-containing region, which has been reported to undergo modifications such as phosphorylation and methylation, has not been sufficiently investigated. Thus, we aimed to explore the functional significance of the Y14 C-terminal region. Deletion or dephosphorylation mimic mutants of the C-terminal region showed a shift in localization from the nucleoplasmic region; in addition, the C-terminal RS repeat-containing sequence itself exhibited the potential for nucleolar localization. Additionally, the regulation of Y14 localization by the C-terminal region was further found to be exquisitely controlled by MAGOH binding. Cumulatively, our findings, which demonstrated that Y14 localization is regulated not only by the previously reported N-terminal localization signal but also by the C-terminal RS repeat-containing region through phosphorylation and MAGOH binding to Y14, provide new insights for the mechanism of localization of short RS repeat-containing proteins.

## Introduction

The formation of ribonucleoprotein particles (RNPs) is crucial for the regulation of RNA splicing, translation, degradation, transport, and localization. In particular, the exon junction complex (EJC), an RNP that forms upstream of exon-exon junctions, is involved in the RNA surveillance system^1–3^. The EJC core is composed of eIF4A3, Y14 (RBM8A), MAGOH, and CASC3 (also called MLN51 or Btz)^4,5^, and functions as a scaffold for various proteins to mediate subsequent events. As a first step in the acquisition of EJC core conformation, eIF4A3, a DEAD-box family protein, binds to RNA through its two RecA domains in an ATP-dependent manner at the spliceosome via the CWC22 escort^6–9^. Next, a Y14/MAGOH heterodimer binds eIF4A3 to inhibit its ATPase activity, enforcing the structure of the RNA-protein complex. This trimeric complex, known as pre-EJC, is fundamentally related to splicing. Pre-EJC construction is followed by CASC3 binding, which confers further stability to the EJC core^4,5,10^.

Among the peripheral EJC components, the UPF family (UPF1, UPF2, UPF3A, and UPF3B) is responsible for classical nonsense-mediated mRNA decay (NMD), which is induced by formation of the EJC core/UPF2/UPF3B and SURF (SMG1, UPF1, eRF1, and eRF3) complexes during the pioneer round of translation^11,12^. In addition, several alternative NMD pathways can degrade RNA^13^. Although the role of these pathways in disease remains to be determined, previous reports have suggested that NMD factors are associated with neuronal dysfunction in diseases such as autism, schizophrenia, and microcephaly^14–16^. Neuronal cells have been reported to use alternative RNA splicing to regulate complicated differentiation processes, which can generate alternatively spliced RNA containing premature termination codons (PTCs)^17^. PTC-containing RNA, which is produced by alternative splicing, is processed and degraded by NMD. Therefore, the regulation of NMD-dependent degradation should be important for neurodevelopment, and defects in EJC factors could induce neurodevelopmental disorders. In addition, the dysfunction of specific EJC factors has been implicated in the development of specific diseases; for example, mutation of the Y14 gene (*RBM8A*), resulting in reduced expression of the encoded protein, has been identified in thrombocytopenia-absent radius syndrome, whereas *EIF4A3* gene mutations were found in Richieri-Costa-Pereira syndrome^18,19^. Thus, to clarify the divergent symptoms that can result from dysregulation of EJC factors, the effects of each EJC factor should be analyzed individually.

With respect to EJC core components, we have especially focused on Y14, which constitutes a 20-kD protein composed of an N-terminal localization signal, C-terminal short RS repeat-containing region, and central RNA recognition motif (RRM) involved in MAGOH binding. In previous studies, we demonstrated that the Y14/MAGOH heterodimer regulates the cell cycle by controlling centrosome duplication; accordingly, knockdown of these factors induced centrosomal abnormalities and apoptotic cell death^20,21^. Moreover, MAGOH, a constitutive Y14 binding partner, has been shown to contribute to cyclin-dependent kinase regulation and cell proliferation^22^. Therefore, Y14 and MAGOH might play a significant role in cellular growth. In cells, Y14 predominantly localizes to the nucleoplasm, especially at the perispeckle^23^, a peripheral region of nuclear speckles that stores various splicing factors required for efficient RNA splicing. However, the function of Y14/MAGOH, including the role of the N-terminal nuclear export signal or how Y14/MAGOH localizes to perispeckles, has not been completely deciphered. Especially, limited information is available regarding the role of the C-terminal short RS repeat-containing sequence of Y14.

RS, arginine/glycine (RG), or arginine/glycine/glycine (RGG) repeats are commonly found in splicing factors^24^. These have primarily been identified in eukaryotes, especially in higher organisms, and are suggested to have been acquired during evolution for divergent genetic regulation^24^. In particular, splicing factor SR proteins comprise a well-known protein family that harbors an RS domain, which is composed of long RS repeats. RS repeats of several SR proteins have been reported to assist in localization to nuclear speckles, splicing activation, and protein-protein/protein-RNA interactions^25–27^. In addition, these motifs have been reported to be phosphorylated by the SRPK protein kinase family^28^. Moreover, the protein phosphatase PP1 has been shown to modulate the phosphorylation status of the RS domain^29^. Notably, these phosphorylation and dephosphorylation events regulate localization or structural changes. Furthermore, the SRPK family is also known to possess the ability to phosphorylate the C-terminal 166 and 168 serine residues of Y14^30,31^. However, the role of Y14 phosphorylation remains poorly understood.

In this study, we investigated the C-terminal sequence of Y14 to reveal its role. The results demonstrated that the Y14 C-terminal short RS repeat-containing sequence functions in modulating the cellular localization of Y14.

## Results

### The C-terminal amino acid sequence of Y14 contributes to nuclear localization in HeLa cells

**Figure 1:**
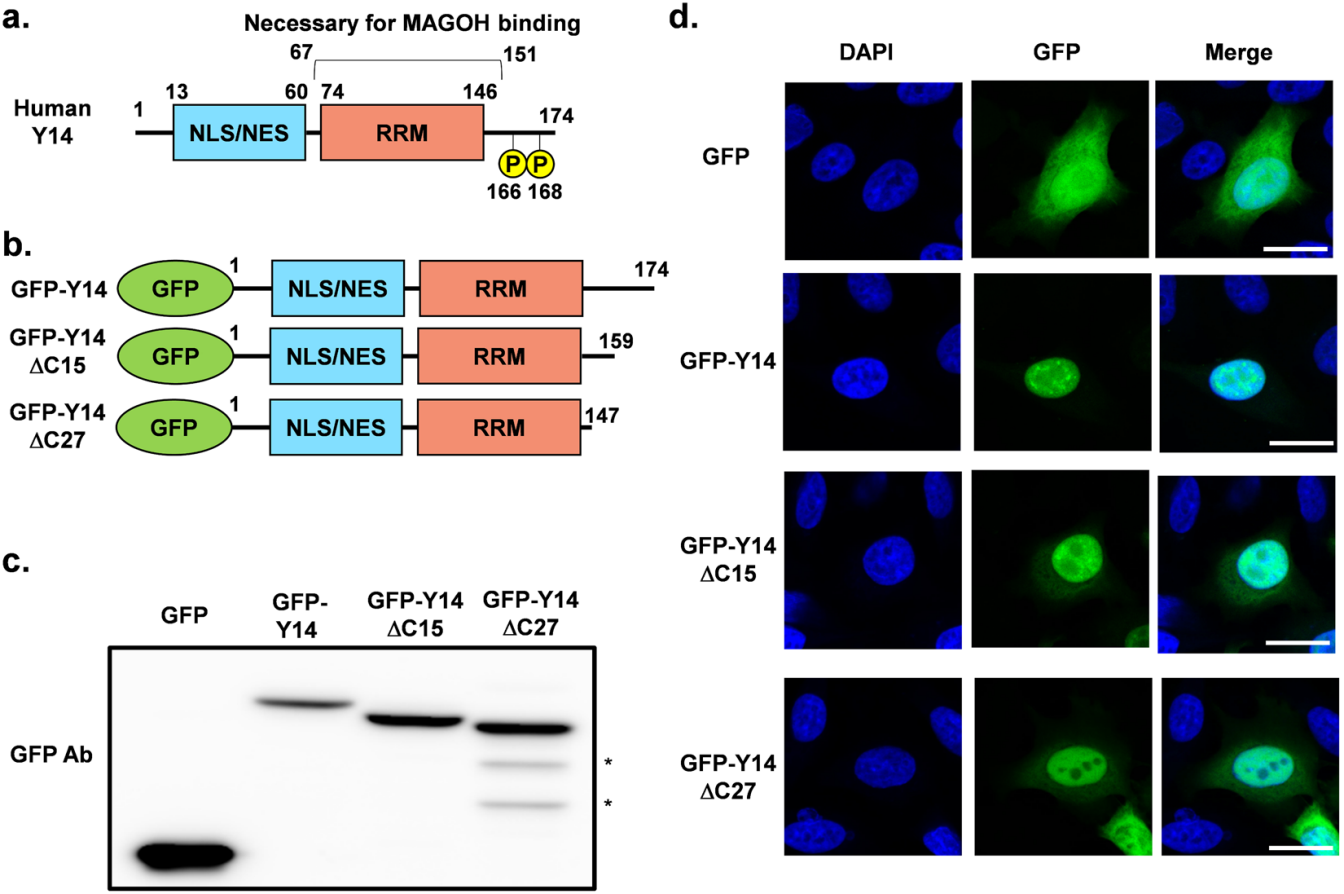
(**a**) Y14 structure indicating the position of previously reported localization signal (NES/NLS), RNA-recognition motif (RRM), and phosphate group (P). (**b**) Structure of Y14 and C-terminal deletion mutants. (**c**) Expression of transfected GFP-Y14 and C-terminal deletion mutants was detected by western blotting with an anti-GFP antibody. Asterisk indicates non-specific bands. The cropped blots are used in the figure. The membranes were cut prior to exposure so that only the portion of gel containing the desired bands would be visualized. Full-length blots are shown in Supplementary Fig. S10. (**d**) Localization of GFP-Y14 and its mutants was observed by fluorescence microscopy (green). DNA existing in the nucleus was stained by DAPI (blue). Bar indicates 20 μm.

First, we focused on the C-terminal RS repeat-containing sequence of Y14 because it is conserved in vertebrates; in addition, serine residues are phosphorylated in several species, although invertebrates such as *Drosophila* or *Caenorhabditis elegans* do not have these regions (Supplementary Fig. S1). To analyze the role of the C-terminal region, we used green fluorescent protein (GFP)-tagged Y14 and C-terminal region-deleted mutants (Y14ΔC15, Y14ΔC27). Y14ΔC15 mutants, lacking the 15 C-terminal amino acid residues, were devoid of phosphorylation sites but retained their MAGOH binding sequence, similar to Y14 of *Drosophila*. Y14ΔC27 was further lacking 12 C-terminal amino acids, compared to Y14ΔC15, resulting in loss of MAGOH binding; however, Y14ΔC27 retained its putative RRM (Fig. 1a,b). Subsequently, expression of these proteins was confirmed by western blotting (Fig. 1c, Supplementary Fig. S2). Based on the fluorescence of overexpressed tagged proteins in HeLa cells, C-terminal deleted mutants exhibited diminished localization to some nuclear regions not stained by DAPI; this was suggested to possibly represent the nucleoli (Fig. 1d, Supplementary Figs S3 and S4). In addition, C-terminal deleted mutants showed a slight increase in leakage to the cytoplasmic region, as compared to that with GFP-Y14. There was a difference in terms of localization between endogenous Y14 and GFP-Y14, as endogenous Y14 is not known to localize to the nucleoli, whereas GFP-Y14 was shown to weakly diffuse to the nucleolar region. However, the C-terminal region of Y14 was suggested to function in nuclear localization. In addition, the MAGOH-binding region was also suggested to be important for nuclear retention based on differences between Y14-ΔC15 (which retains the MAGOH-binding site) and Y14-ΔC27 (which lacks the 4 amino acids necessary for MAGOH binding). These data indicate that the C-terminal short RS repeat-containing sequence enhances nuclear localization.

### Phosphorylation of C-terminal serine residues of Y14 regulates localization between the nucleoplasm and nucleoli

**Figure 2:**
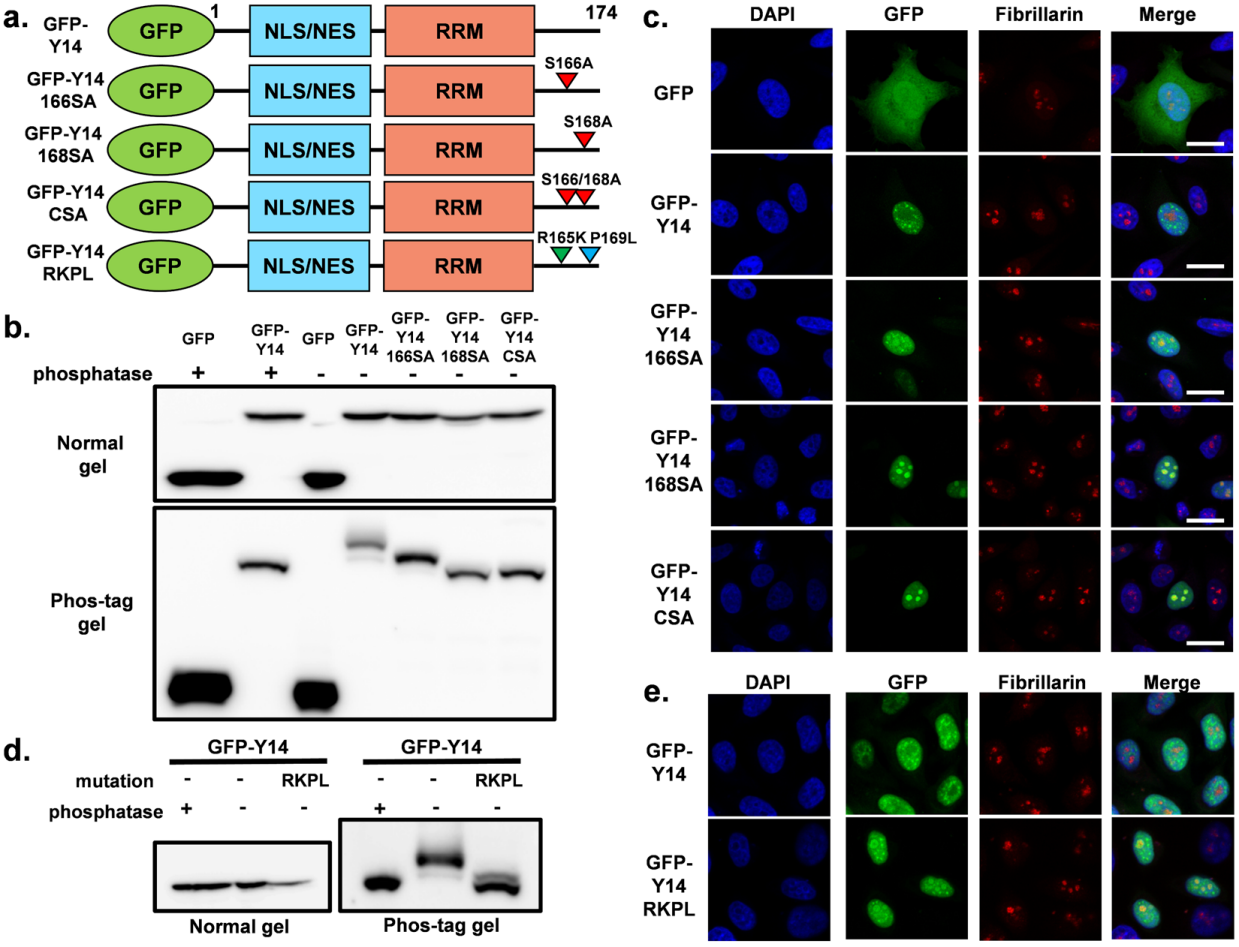
(**a**) Structure of GFP-tagged Y14 serine-alanine substitution mutants and the RKPL mutant, which mimics dephosphorylated Y14. (**b**) Expression of transfected GFP-Y14 and serine-alanine substitution mutants was detected by western blotting with an anti-GFP antibody (upper panel). Phosphorylation status of Y14 and mutants was analyzed by separation using phos-tag gels (bottom panel). The samples for SDS-PAGE and phos-tag SDS-PAGE derive from the same experiment. The cropped blots are used in the figure. The membranes were cut prior to exposure so that only the portion of gel containing the desired bands would be visualized. Full-length blots are shown in Supplementary Fig. S10. (**c**) Localization of GFP-Y14 and its associated mutants was observed by fluorescence microscopy (green). Fibrillarin and nuclear DNA are shown as red and blue, respectively. Bar indicates 20 μm. (**d**) Expression of transfected GFP-Y14 and RKPL mutant was detected by western blotting with an anti-GFP antibody (left panel). Phosphorylation status of Y14 and mutants was analyzed by separation using phos-tag gels (right panel). (**e**) Localization of GFP-Y14 and the RKPL mutant was observed by fluorescence microscopy (green). Fibrillarin and nuclear DNA are shown as red and blue, respectively. Bar indicates 20 μm.

We next explored the effect of serine phosphorylation of the C-terminal RS repeat-containing region of Y14 on nuclear localization. From previous results, it is known that human Y14 is phosphorylated on residues 166 and 168. Thus, we constructed a GFP-Y14 expression vector with serine to alanine mutations at 166 (Y14 166SA), 168 (Y14 168SA), or both (Y14 CSA) (Fig. 2a). Although we added a GFP tag to the N-terminus of Y14, GFP-Y14 and its mutants exhibited the same phosphorylation pattern shown in a previous study^31^ (Fig. 2b, Supplementary Fig. S2). With the 168SA and CSA mutants, co-localization (based on merged fluorescence signal) with the nucleolar protein fibrillarin was more clearly observed. Furthermore, the 166SA mutant weakly localized to the nucleoli (Fig. 2c, Supplementary Fig. S3). These results were consistent with the differential phosphorylation status of Y14. To avoid artificial effects from serine to alanine mutation, we further constructed a non-phosphorylation mimic mutant without the alteration of serine residues (Y14 RKPL: arginine to lysine at 165, proline to leucine at 169) (Fig. 2a). Based on phos-tag gel electrophoresis, the RKPL mutant was predominantly dephosphorylated, similar to 168SA or CSA, whereas subtle bands representing the phosphorylated form could be detected (Fig. 2d, Supplementary Fig. S2). Consistent with the phosphorylation status, the GFP-Y14 RKPL mutant mainly localized to the nucleoli, similar to GFP-Y14 168SA or CSA mutants (Fig. 2e, Supplementary Fig. S3). In addition, nucleolar localization of the dephosphorylation mimic mutant was verified using a GST-tagged variant (Supplementary Fig. S5). Additionally, whereas dephosphorylation mimic mutants showed nucleolar localization, these GFP-tagged mutants maintained merged localization with SRSF2, presumably localization at perispeckle^23^. (Supplementary Fig. S6). Collectively, dephosphorylated Y14 was suggested to localize to the nucleoli and the phosphorylation of C-terminal serine residues was shown to prevent the nucleolar localization of Y14.

### Confirmation of fluorescence results using fractionation of cells expressing GFP-tagged Y14 and mutants

**Figure 3:**
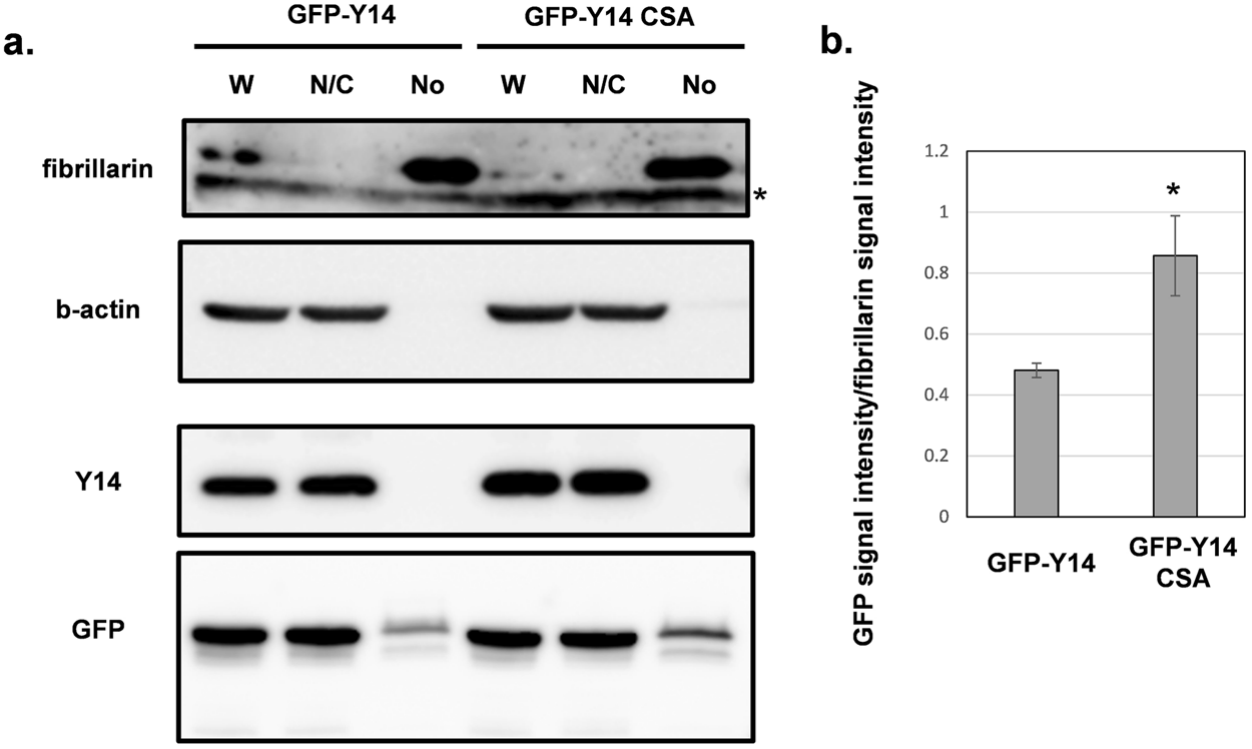
(**a**) Representative western blot of fractionated samples. The cropped blots are used in the figure. The membranes were cut prior to exposure so that only the portion of gel containing the desired bands would be visualized. The samples of fractionation derive from the same experiment and the blots were processed in parallel. Full-length blots are shown in Supplementary Fig. S10. (**b**) Bar-graph of associated quantification. Each bar shows the value derived from GFP intensity of nucleolar fraction/fibrillarin intensity of nucleolar fraction. *P < 0.05. P values were calculated by performing a t-test.

Next, to verify the localization of GFP-Y14 and dephosphorylation mimic mutants, based on fluorescent observations, we performed nucleolar fractionation. Transfected cells were fractionated to obtain a nucleocytoplasmic compartment and a nucleolar compartment, and existence of proteins was detected by western blotting (Fig. 3a,b). Whereas it was shown that nucleolar purification might affect the localization of GFP-Y14 and the GFP-Y14 CSA mutant since the difference between GFP-Y14 and the GFP-Y14 CSA mutant was not so clear in comparison with that of fluorescent observation under microscope, the GFP-Y14 CSA mutant exhibited significantly stronger expression in the nucleolar fraction compared to that for GFP-Y14 (P < 0.05). This result was consistent with observations in Fig. 2. As an experimental control of nucleolar fractionation with protein overexpression, we performed the same experiment with another RNA binding motif-containing protein, SRSF3. Upon expression of GFP-tagged SRSF3, both endogenous and GFP-tagged SRSF3 showed similar band patterns (Supplementary Fig. S7).

### The C-terminal region of Y14 contains a nucleolar localization signal that is regulated by phosphorylation

**Figure 4:**
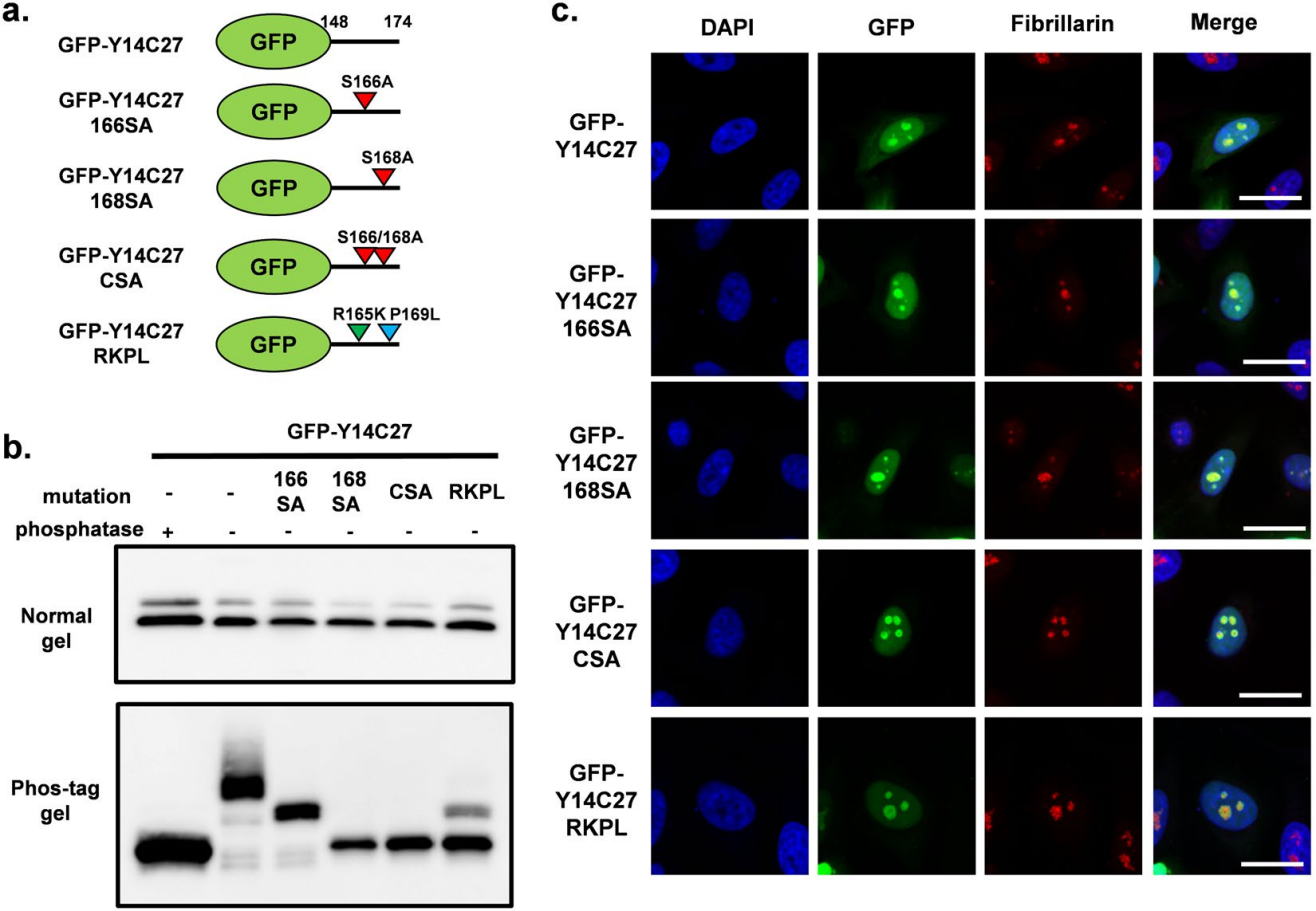
(**a**) Structure of GFP-tagged C-terminal fragment of Y14 or its corresponding mutants. (**b**) Expression of transfected GFP-Y14 and RKPL mutant was detected by western blotting with an anti-GFP antibody (upper panel). Phosphorylation status of Y14 and mutants was analyzed by separation using phos-tag gels (bottom panel). The samples for SDS-PAGE and phos-tag SDS-PAGE derive from the same experiment. The cropped blots are used in the figure. The membranes were cut prior to exposure so that only the portion of gel containing the desired bands would be visualized. Full-length blots are shown in Supplementary Fig. S10. (**c**) Localization of GFP-Y14C27 and mutants was observed by fluorescence microscopy (green). Fibrillarin and nuclear DNA are shown as red and blue, respectively. Bar indicates 20 μm.

The above findings suggested that the dephosphorylated Y14 C-terminal RS repeat-containing region might represent a strong nucleolar localization signal. Thus, to clarify the role of this region, we generated a construct comprising the GFP-tagged Y14 C-terminal 27 amino acid residues (GFP-Y14C27) and the corresponding SA or RKPL mutants (Fig. 4a). Consistent with full length Y14 and mutants, a gel shift was observed for GFP-Y14C27 and the GFP-Y14C27 166SA mutant, but not for 168SA, CSA, or RKPL, after phos-tag gel electrophoresis (Fig. 4b, Supplementary Fig. S2). However, unexpectedly, GFP-Y14C27 and all corresponding mutants strongly localized to the nucleoli, although there was a slight difference in signal distribution at the cytoplasmic region between GFP-Y14C27 and the mutants (Fig. 4c, Supplementary Fig. S3). Thus, the Y14 C-terminal RS repeat-containing region was identified as a nucleolar localization signal which was slightly regulated by phosphorylation status.

### MAGOH binding disrupts nucleolar localization

**Figure 5:**
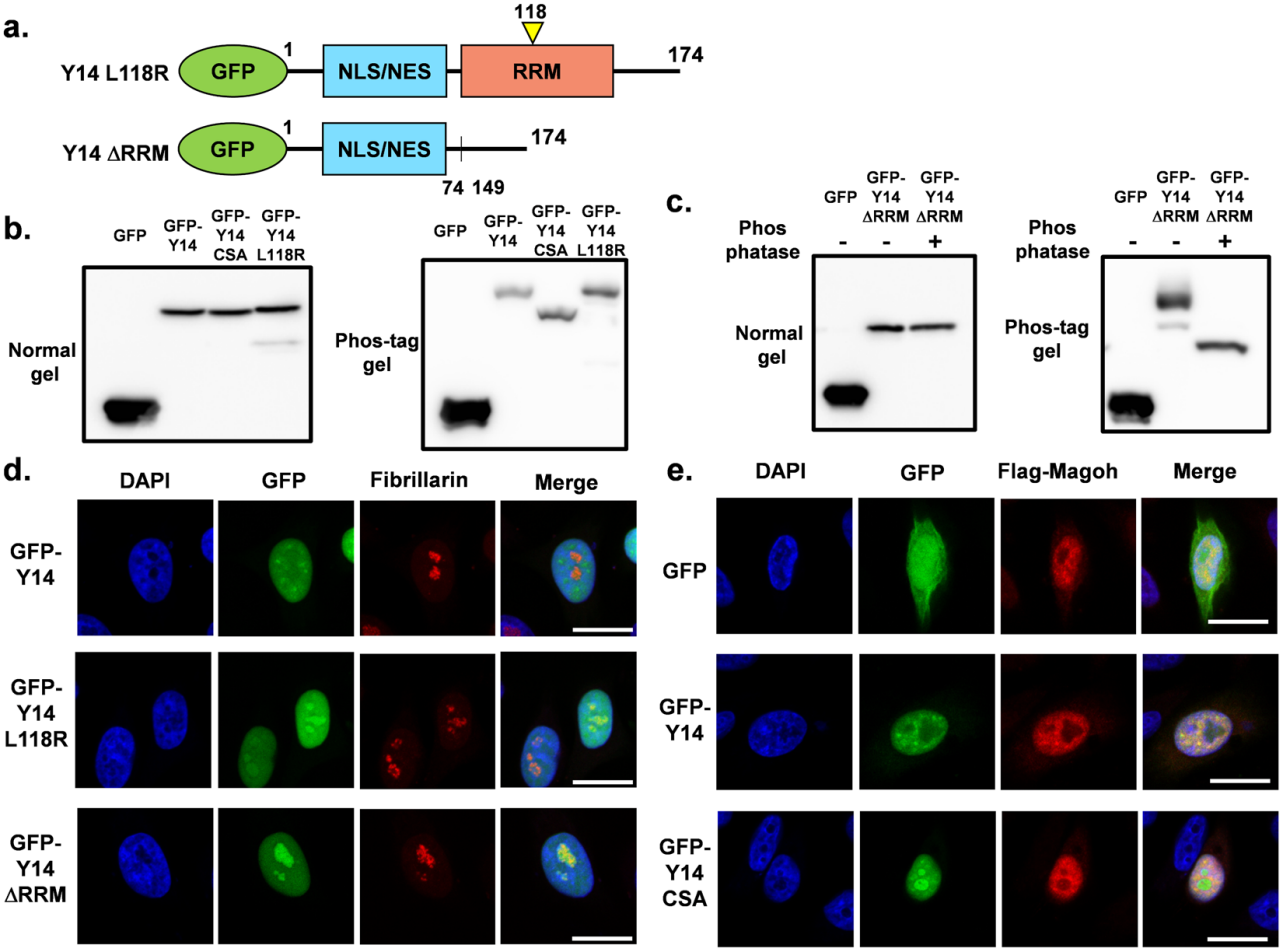
(**a**) Structure of GFP-tagged Y14 mutants that lack MAGOH binding ability. (**b**) Expression of the transfected GFP-Y14 L118R mutant was detected by western blotting with an anti-GFP antibody (left panel). Phosphorylation status of GFP-Y14 L118R was analyzed by separation using phos-tag gels (right panel). The samples for SDS-PAGE and phos-tag SDS-PAGE derive from the same experiment. The membranes were cut prior to exposure so that only the portion of gel containing the desired bands would be visualized. Full-length blots are shown in Supplementary Fig. S10. (**c**) Expression of the transfected GFP-Y14 ΔRRM mutant was detected by western blotting with an anti-GFP antibody (left panel). Phosphorylation status of GFP-Y14 ΔRRM was analyzed by separation using phos-tag gels by comparing with phosphatase-treated GFP-Y14 ΔRRM (right panel). The cropped blots are used in the figure. The samples for SDS-PAGE and phos-tag SDS-PAGE derive from the same experiment. The membranes were cut prior to exposure so that only the portion of gel containing desired bands would be visualized. Full-length blots are shown in Supplementary Fig. S10. (**d**) Localization of GFP-Y14, L118R, and ΔRRM mutants was observed by fluorescence microscopy (green). Fibrillarin and nuclear DNA are shown as red and blue, respectively. Bar indicates 20 μm. (**e**) GFP-Y14 and CSA mutant were observed by fluorescence microscopy (green). Localization of Flag-MAGOH and nuclear DNA is shown as red and blue, respectively. Structures of mutants are shown in Fig. 2. Bar indicates 20 μm.

Based on the localization analysis of the Y14 C-terminal RS repeat-containing region, it was suggested that nucleolar localization of Y14 might not be regulated solely by the Y14 C-terminal RS repeat-containing region and its phosphorylation. Therefore, we decided to investigate the effect of MAGOH binding and the N-terminal localization signal on nucleolar localization. To determine the role of the heterodimeric Y14-binding protein MAGOH and the N-terminal localization signal, we utilized Y14 mutants (GFP-Y14 L118R with a leucine-to-arginine substitution at amino acid residue 118 and GFP-Y14ΔRRM) that cannot bind MAGOH (Fig. 5a)^32^. Based on phos-tag gel electrophoresis, the L118R mutant was predominantly phosphorylated, similar to GFP-Y14 (Fig. 5b, Supplementary Fig. S2). Furthermore, the phosphorylation of the Y14ΔRRM mutant was confirmed by phosphatase treatment (Fig. 5c). Both GFP-Y14 L118R and GFP-Y14ΔRRM localized to the nucleoli, similar to dephosphorylation mimic mutants (Fig. 5d, Supplementary Fig. S3). In addition, it was clarified that the N-terminal localization signal of Y14 has no effect on nucleolar localization, as GFP-Y14ΔRRM, which retains the N-terminal localization signal, could strongly localize to nucleoli. Subsequently, to clarify MAGOH localization under the expression of nucleolar-localized Y14, we co-transfected Flag-MAGOH and GFP-Y14 mutants. Based on immunostaining results, Flag-tagged MAGOH did not exhibit nucleolar localization, whereas Flag-MAGOH and GFP-Y14 mutants co-localized to the nucleoplasm but not the nucleolar compartment (Fig. 5e). Together, these findings revealed that nucleolar localization of Y14 is restricted by phosphorylation of C-terminal serine residues and interaction with MAGOH.

## Discussion

Since the identification of splicing-dependent RNA binding protein around the exon-exon junction^4^, various EJC factors have been found and shown to be associated with processes related to mRNA metabolism, such as translation, degradation, transport, and localization. Although some EJC factors were reported to affect e.g., neurodevelopment, cell growth, and differentiation^14–16,22^, differences regarding the mechanisms through which disruption of EJC factors induces various disorders remains unclear. Previously, the signal sequence of Y14 for localization to nuclear or cytoplasmic regions was reported to reside in the N-terminal part of the protein^32^. This region was shown to be important for Y14 movement to the nucleus, as this protein constitutes a core EJC factor that is deposited on the RNA during splicing in the nucleus, and plays significant roles in various RNA metabolic processes such as transport and translation. Whereas the N-terminal localization signal is sufficient for nuclear localization, the present study showed that Y14 contains another signal sequence that modulates nuclear localization in human cells.

In the present study, we focused on the C-terminal short RS-repeat sequence of Y14. Previously, RS repeats in Y14 were reported to be phosphorylated and methylated, and to bind TAP, which is an mRNA factor that modulates transport from the nucleus to the cytoplasm^30^. In addition, Y14 C-terminal short RS repeats were shown to be associated with decapping and inhibition of decapping by disrupting the dephosphorylation of Y14 lead to p-body accumulation^33^, presumably affecting mRNA stability. Although we could not detect non-phosphorylated Y14 in cultured cells and Y14 showed similar localization patterns as the phosphorylation mimic mutant (Supplementary Fig. S8), a significant difference in phosphorylation or methylation status may exist depending on the conditions of the experiments. In the present investigation, we used exogenous overexpression to express a Y14 dephosphorylation mimic based on the difficulty in dephosphorylating endogenous Y14 in HeLa cells (Supplementary Fig. S2). Y14 is strongly phosphorylated in these cells and the associated mechanism is not fully understood, although the SRPK family has been suggested to have the capacity to phosphorylate Y14. In our experimental conditions, dephosphorylation mutants of full length Y14 localized to the nucleoli, whereas loss of the C-terminal RS repeat-containing region of Y14 resulted in apparent removal from the nucleoli along with cytoplasmic leakage (Figs 1, 2 and 3). Furthermore, full length Y14, in addition to a dephosphorylation mimic mutant, was shown to localize to perispeckles, similar to that observed with SR proteins, although we could not differentiate perispeckles from nuclear speckles based on the settings of our microscope (Supplementary Fig. S6). In contrast, the C-terminal region of Y14 showed apparent nucleolar localization without localization to nuclear speckles. Moreover, although subcellular localization of Y14 was regulated by the RS repeat-containing region and its phosphorylation, these results differed from the localization mechanism that was previously reported for the long RS-repeats of SR proteins^25^ (Fig. 4). In addition, the Y14 C-terminal RS repeat-containing region could serve as a supporting sequence through its association with the MAGOH binding region, as virtually no wild-type Y14 localized to the nucleoli, whereas MAGOH-binding-incompetent mutants showed nucleolar localization (Fig. 5). Finally, although our findings clearly demonstrated differences in localization depending on the RS repeat-containing region and its phosphorylation, these results might be affected by exogenous overexpression of tagged proteins to some extent since overexpression efficiency of Y14 and experimental control SRSF3 was different. (Supplementary Fig. S7). Thus, further investigation should be performed to understand the role of the RS repeat-containing region in cells with endogenous Y14 expression levels.

Although we could not detect dephosphorylated Y14 in cells from the results of phos-tag gel electrophoresis of GFP-Y14, consistent with previous results^31^, it is possible that dephosphorylation of Y14, inducing a shift in localization to various nuclear bodies, might occur under specific physiological stresses such as UV exposure, which induces SR and SR-related proteins to redistribute to DNA damage-induced nucleolar organizing region-associated patches (d-NAPs), or heat shock, which causes HSF1 to form nuclear stress bodies^34,35^. The RS repeat-containing region is not present in Y14 homologues such as tsunagi of *Drosophila melanogaster* or rnp-4 of *C. elegans*, whereas the C-terminal RS-repeats are preserved in vertebrates (Supplementary Fig. S1). It is possible that Y14 acquired the RS repeat-containing region during evolution to strictly regulate Y14 distribution to the nucleoplasm and enhance the efficiency of localization to perispeckles to construct the EJC (Supplementary Fig. S9) or to accurately control localization under physiological stress. Furthermore, this localization regulatory mechanism might be applicable in plants as a GFP-tagged Arabidopsis Y14 homologue showed nucleolar localization^36^. In addition to the function of RS-repeats in localization, including the Y14 nucleoplasmic retention mechanism, some RS-repeats have other roles such as splicing activation or histone binding^27,37,38^. Therefore, the C-terminal short RS repeat-containing region of Y14 might have additional functional relevance as such regions have various roles in protein function, independent of the similarity of phosphorylation pattern or sequence.

Recently, several studies have reported the relationship between C9ORF72 and amyotrophic lateral sclerosis/frontotemporal dementia^39^. Expansion of hexanucleotide (GGGGCC) repeats in C9ORF72 causes the production of dipeptide repeats including glycine/arginine (GR) and proline/arginine (PR). Both GR and PR repeats have been reported to localize to the nucleolus and induce nucleolar stress^40^. Moreover, these dipeptide repeats are known to interact with splicing factors such as U2 snRNP or SR proteins, which are implicated in splicing defects^41,42^. From the viewpoint of localization, GR and PR should function similarly to short C-terminal Y14 RS-repeats as these all strongly exhibit nucleolar localization. Thus, the toxicity of superfluously dephosphorylated RS-repeats was suspected to occur in the same manner as that induced by GR or PR dipeptide repeats. Therefore, the regulation of RS-repeats by phosphorylation or protein binding adjacent to RS-repeats might be important for the cellular function of RS-repeat-containing RNA binding proteins under conditions of non-physiological stress. Furthermore, dysregulation of Y14 may be fundamentally associated with ALS, as Y14 has been reported to interact with STAT3, the activation of which has been observed in the spinal cord of patients with sporadic ALS^43,44^.

In conclusion, the RS repeat-containing region of the C-terminal sequence of Y14 plays a role in nucleoplasmic localization. During this regulation, the phosphorylation of Y14 and binding to MAGOH allows Y14 to avoid transfer to the nucleoli, while retaining its ability to reside in the nucleoplasm. By focusing on the C-terminal region we thus clarified the mechanism of Y14 localization; however, further investigation should be performed to reveal the functional relevance of the strict regulation of nucleoplasmic localization.

## Materials and Methods

### Cell culture

The HeLa cell line was acquired from RIKEN Bioresource Center and was cultured in Dulbecco’s modified Eagle’s medium (Wako Pure Chemical Industries) with 4.5 g/L glucose supplemented with 10% fetal bovine serum (Sigma-Aldrich), 100 mg/mL penicillin, and 100 U/mL streptomycin (Wako) at 37 °C in 5% CO_2_.

### Vector construction

The Y14-encoding sequence, inserted in the pReceiver-M29 vector, was purchased from Genecopoeia. The Y14 serine-to-alanine mutant vector was constructed as previously described^31^. Each mutation or deletion in the C-terminal sequence of Y14 was generated using PrimeSTAR Max DNA Polymerase (TaKaRa Bio) or the QuikChange Site-Directed Mutagenesis Kit (Agilent Technologies) according to the manufacturer’s instructions. For the construction of a GFP-tagged Y14 C-terminal fragment expression vector, or to generate mutations in GFP-tagged Y14, The DNA sequence of the C-terminal 27 amino acid residues were amplified by polymerase chain reaction from full length Y14 and mutants, and inserted into pReceiver-M29 using the infusion system or restriction enzyme digestion method with NspV and XhoI. Primers were obtained from Eurofins Genomics and the sequences of the primers were: Y14(148-)-F: CAGATCTTCGAACCATGTG GTGTTTTGTTCGG, Y14(-174)-R: GGCGGCCTCGAGCTAGCGACGTCTCCGGTC, Y14(1-)-F: CAGATCTTCGAACCATGGCGGACGTGCTAG, Y14(169PL)-R: CATGG TCTCGAGTCAGCGACGTCTCCGGTCTAGAC, Y14(165RK)-F: GGTGGCCGAAG AAAGAGCAGAAGTCCAGACCGG, Y14(165RK)-R: CCGGTCTGGACTTCTGCT CTTTCTTCGGCCACC, Y14(-159)-R: GTACTGCTCGAGCTACCTCTTGCCTTTTG GTG, Y14(-147)-R: GTACTGCTCGAGCTAGTCAACGCTGATGGGCTG, Y14 (ΔRRM)-F: CTGGATTTGTTTTGTTCGGGGTCCA, Y14(ΔRRM)-R: ACAAAACAA ATCCAGCCTTCAACAGA, Y14(L118R)-F: TATACTCGAGTTGAATATGAAACAT AC, Y14(L118R)-R: TTCAACTCGAGTATACCCCTTCAGATA, pReceiver-M29-F: TAGCTCGAGTGCGGCCGCA, and pReceiver-M29-R: CATGGTTCGAACGCTTTC. Vectors were confirmed by sequencing using the BigDye Terminator v3.1 Cycle Sequencing Kit (Thermo Fisher Scientific) and a 3500xL Genetic Analyzer (Thermo Fisher Scientific). The Flag-MAGOH expression vector was purchased from Genecopoeia and directly used for transfection.

### Localization analysis

Fluorescence observation was performed as previously described^21^ with minor modifications. Briefly, HeLa cells were seeded on a 12 mm round cover glass. The next day, cells were transfected with vectors that encoded GFP-Y14, its corresponding mutants, or Flag-MAGOH using Lipofectamine 3000 (Thermo Fisher Scientific). Two days after transfection, cells were washed with phosphate buffered saline (PBS) and fixed for 20 min at room temperature in 2.5% paraformaldehyde/PBS. After fixation, cells were washed with PBS and permeablized with 0.1% triton-X/PBS for 10 min. Next, fixed and permeablized cells were blocked with 5% skim milk/PBS (Wako) for 1 h. Cells were then stained with mouse anti-fibrillarin (Abcam) or mouse anti-Flag (Sigma-Aldrich) antibodies diluted in 0.5% skim milk. Following binding of the primary antibody, mouse anti-fibrillarin or mouse anti-Flag (Sigma-Aldrich) antibodies were detected using Alexa Fluor 594-conjugated secondary antibody (Thermo Fisher Scientific) diluted in blocking buffer containing DAPI (final concentration: 10 μg/mL; Thermo Fisher Scientific). Labeled cells were washed with PBS and embedded in Prolong Gold anti-fade reagent (Thermo Fisher Scientific) on the slide glass. For the deletion mutants, cells were embedded in Prolong Gold anti-fade reagent with DAPI after fixation and PBS washing. Then, cells were observed using the LSM710 (Carl Zeiss). Captured images were processed with ZEN 2012 (blue edition) (Carl Zeiss).

### Nucleoli purification

Nucleoli purification was performed according to a previously published protocol^45^. HeLa cells were cultured in a 10-cm dish to subconfluency and transfected with the plasmid vectors. One day after transfection, cells were washed with pre-cooled solution I (0.5 M sucrose, 3 mM MgCl_2_ with a protease inhibitor cocktail (Roche)) at −20 °C. Cells were then scraped from the dish and collected into a tube. The cell suspension was sonicated on ice until the cytoplasmic region was destroyed. The sonicated cell suspension was then overlaid on pre-cooled solution II (1.0 M sucrose, 3 mM MgCl_2_ with a protease inhibitor cocktail) and centrifuged at 1,800 × *g* for 5 min at 4 °C. After centrifugation, the supernatant was collected as the nucleocytoplasmic fraction and the pellet was suspended in solution I as the nucleolar fraction.

### SDS-PAGE/western blot analysis

For the elucidation of phosphorylation status, phos-tag SDS-PAGE, which is capable of separating phosphorylated and non-phosphorylated proteins based on phosphorylation levels by capturing phosphate residues, was used. Phos-tag gels were purchased from Wako. Cell lysates or fractionated samples were used for western blotting. To obtain dephosphorylated protein as a completely dephosphorylated control, lambda protein phosphatase (New England Biolabs) treatment was performed prior to SDS-PAGE. Each fraction or cell lysate was mixed with Laemmli sample buffer (Bio-Rad), resolved on SDS-PAGE or phos-tag SDS-PAGE, and transferred to PVDF membranes. Protein-bound membranes were blocked with 5% skim milk buffer and incubated with primary antibody diluted in 0.5% skim milk buffer. Mouse anti-beta-actin antibody (1:5000, Sigma-Aldrich), rabbit anti-Y14 antibody (1:2000, Sigma-Aldrich), goat anti-GFP antibody (1:2000, Santa Cruz Biotechnology), and mouse anti-fibrillarin antibody (1:1000) were used as the primary antibody. Binding of primary antibody was detected with horseradish peroxidase-conjugated anti-mouse IgG, anti-goat IgG, and anti-rabbit IgG antibodies (Agilent Technologies). The membrane was subsequently washed and developed with ImmunoStar Zeta chemiluminescent reagents (Wako) and chemiluminescence was visualized using the LAS4000 (Fujifilm).

### Statistical analysis

Data were analyzed using a Student’s t test. Values of P < 0.05 indicated statistically significant differences.

## Electronic supplementary material

Supplementary information
