# Supplementary material for: C-terminal short arginine/serine repeat sequence-dependent regulation of Y14 (RBM8A) localization
Source: Sci Rep. 2018 Jan 12;8:612. doi: 10.1038/s41598-017-18765-1 (PMC5766523; doi:10.1038/s41598-017-18765-1)
Supplement: Supplementary file 1 — Supplementary information [file 41598_2017_18765_MOESM1_ESM.pdf]

Supplementary Information

C-terminal short arginine/serine repeat sequence-dependent regulation of Y14 (RBM8A) localization

Takanori Tatsuno, Yasuhito Ishigaki

Fig. S1. Homology of Y14 C-terminal amino acids between various Y14 homologues.
This figure shows the C-terminal region of Y14 from the edge of the MAGOH-binding site to the end of the Y14 amino acid sequence. Arrowhead indicates phosphorylated serine residues in human cells. For vertebrates, the C-terminal amino acid sequence of Y14 is almost completely conserved from humans to zebrafish. However, this region is not conserved in invertebrates such as flies or nematodes, especially regarding the phosphorylatable serine residues.

Fig. S2. Comparison of expression between overexpressed and endogenous protein.
Relative expression levels of overexpressed Y14 including mutants and endogenousY14 were detected by western blotting. Black arrowhead shows GFP-tagged proteins and black outlined arrowhead shows endogenous proteins. Although the anti-Y14 antibody cannot detect the C-terminal RS repeat-containing region, the amounts of overexpressed GFP-Y14C27 and its mutants were larger than those of full length GFP-tagged Y14 or endogenous Y14.

Fig. S3. Low magnification fluorescence image of Y14 and Y14 mutants.
Representative fluorescence images of Y14 and its mutants with multiple numbers of cells. As in the main figure, fibrillarin was detected using an antibody and is shown as red. Nuclear DNA was stained with DAPI and is shown as blue. Bar indicates 50 μm.

Fig. S4. High contrast fluorescence image of Y14 and deletion mutants.
Cytoplasmic differences within Figure 1d in the main document are difficult to differentiate. Therefore, this figure shows the fluorescence image of Y14 and deletion mutants with high contrast to clarify the cytoplasmic differences.

Fig. S5. Localization of GST-tagged Y14 and Y14 CSA mutant.
Localization of GST-tagged proteins was visualized by immunostaining. Fixed, permeablized, and blocked cells were stained with rabbit anti-GST antibody (Medical &

Biological Laboratories) and mouse anti-fibrillarin. Primary antibodies were detected using Alexa Fluor 594-conjugated secondary antibody and Alexa Fluor 488-conjugated secondary antibody diluted in blocking buffer containing DAPI. GST, fibrillarin and nuclear DNA are shown as red, green, and blue, respectively. Bar indicates 20 μm.

Fig. S6. Co-localization of GFP-tagged Y14 and mCherry-SRSF2.
Localization of GFP-tagged Y14 and the Y14 CSA mutant to the peripheral region of nuclear speckles was confirmed by the nuclear speckle marker SRSF2, although perispeckles and nuclear speckles could not be differentiated based on the resolution of our microscope. Nuclear DNA is shown in blue. Bar indicates 20 μm.

Fig. S7. Overexpression of another GFP-tagged RBP in nucleolar fractionation.
(a) As an experimental control for nucleolar fractionation, another RNA binding protein, SRSF3, was used to check for an overexpression effect. Expression amounts of Y14 and SRSF3 proteins were detected by western blotting using rabbit polyclonal anti-Y14 antibody and rabbit polyclonal anti-SRSF3 antibody (Abcam), respectively. Black arrowhead shows GFP-tagged proteins and black outlined arrowhead shows endogenous proteins. (b) Localization of GFP-tagged SRSF3 was shown. Consistent with the previous result[1], GFP-tagged SRSF3 did not exhibit localization to some nuclear regions not stained by DAPI. Bar indicates 20 μm.

Fig. S8. Localization of Flag-tagged Y14 and phosphorylation mimic mutant.
Serine to glutamic acid substitution mutant was used as a phosphorylation mimic mutant. The Y14 CSE mutant has serine-to-glutamic acid substitution at amino acid residue 166 and 168. Localization of Flag-tagged proteins was visualized by immunostaining. Fixed, permeablized, and blocked cells were stained with mouse anti-Flag antibody (Sigma-Aldrich). Primary antibodies were detected using Alexa Fluor 488-conjugated secondary antibody diluted in blocking buffer containing DAPI. Flag and nuclear DNA are shown as green and blue, respectively. Bar indicates 20 μm.

Fig. S9. Suggested graphical model of Y14 localization.
We hypothesize that phosphorylation of Y14 enhances nucleoplasmic retention to effectively deliver the protein to perispeckles for the construction of the exon junction complex. Without phosphorylation, Y14 tends to localize to the nucleoli. Loss of the C-terminal RS-rich region of Y14, which is shown as Y14ΔRS in the figure, leads to weak localization in the cytoplasm. Y14ΔRS is similar to tsunagi or rnp-4, Y14 homologues of

D. melanogaster or *C. elegans*, respectively.

Fig. S10. Original full western blot exposures of the blots shown in the manuscript. The cropped area used in the manuscript is shown by a dotted line frame in the original full western blot exposures. For the western blot of fibrillarin described in Fig. 3a, overexposure of the blot was used in the manuscript, as we could not detect fibrillarin in the whole lysate shown in the blot with short exposure.

Fig. S1.

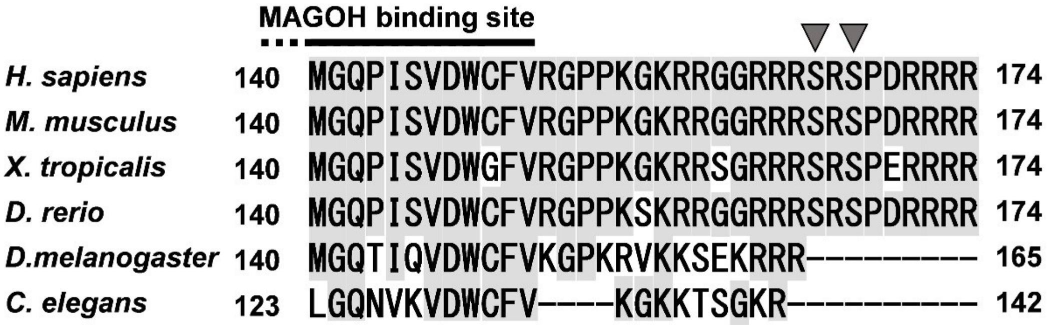

Fig. S2.

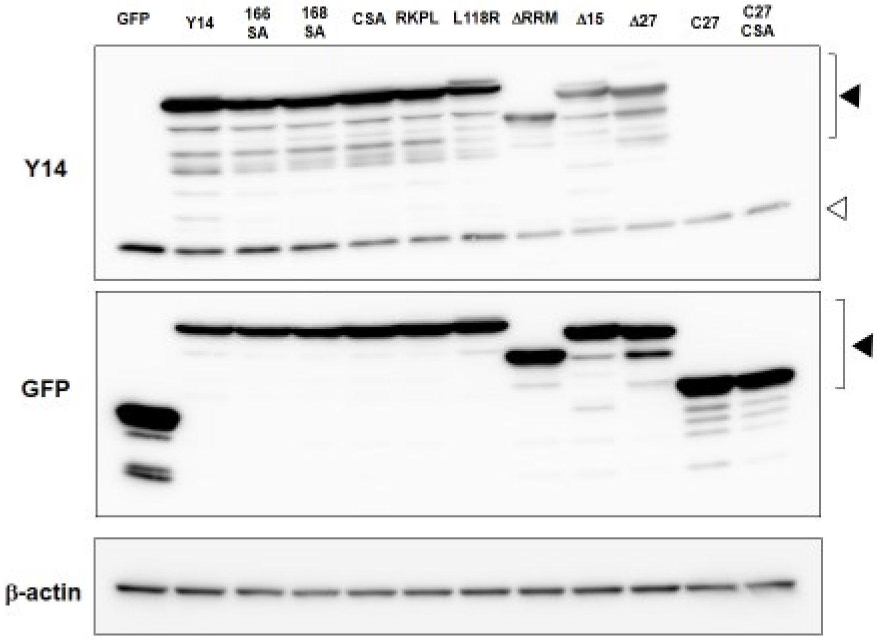

Fig. S3.

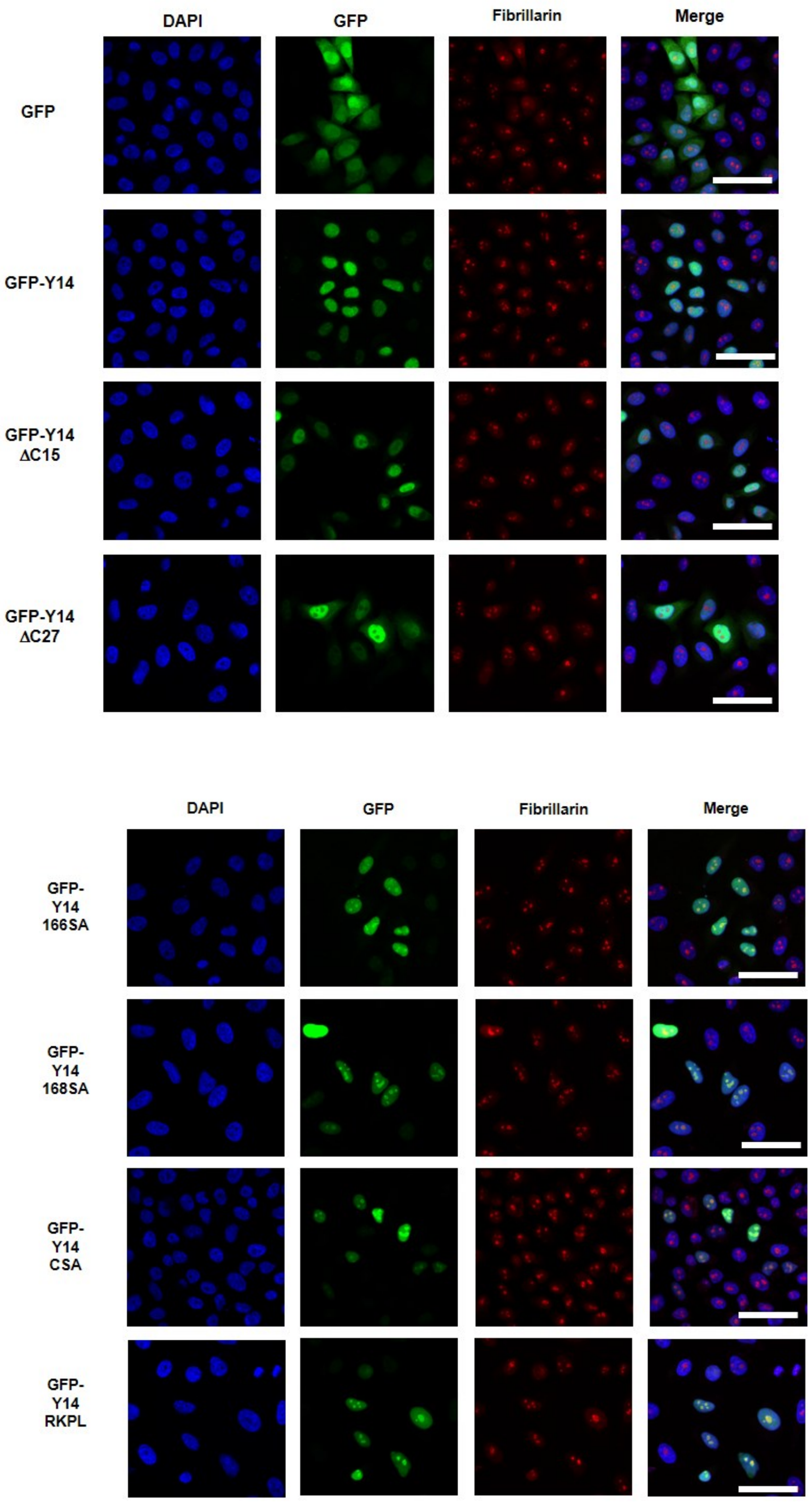
DAPI
GFP
Fibrillarin
Merge
GFP
GFP-Y14
GFP-Y14 ΔC15
GFP-Y14 ΔC27
DAPI
GFP
Fibrillarin
Merge
GFP-Y14 166SA
GFP-Y14 168SA
GFP-Y14 CSA
GFP-Y14 RKPL

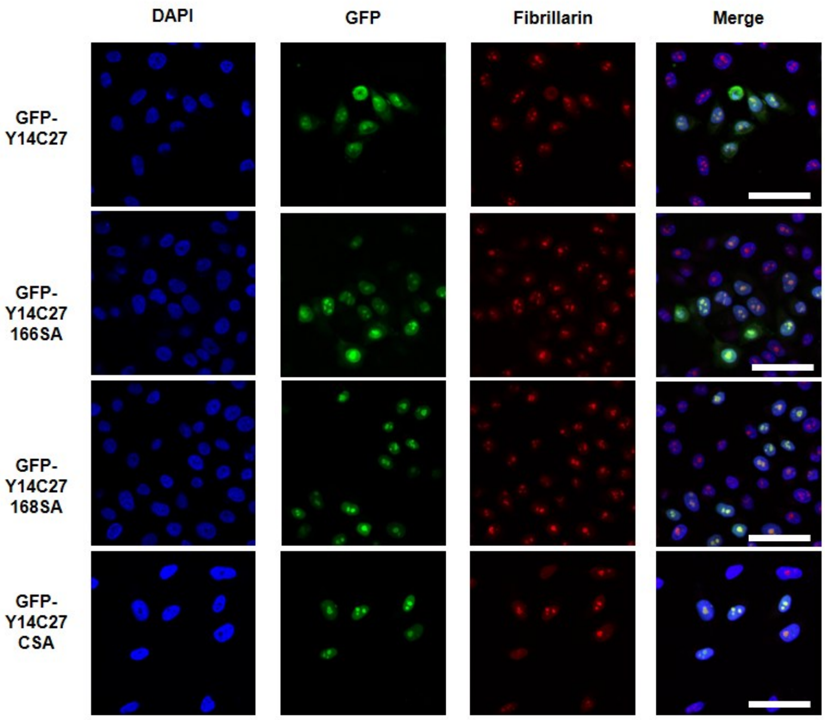
DAPI
GFP
Fibrillarin
Merge
GFP-
Y14C27
GFP-
Y14C27
166SA
GFP-
Y14C27
168SA
GFP-
Y14C27
CSA

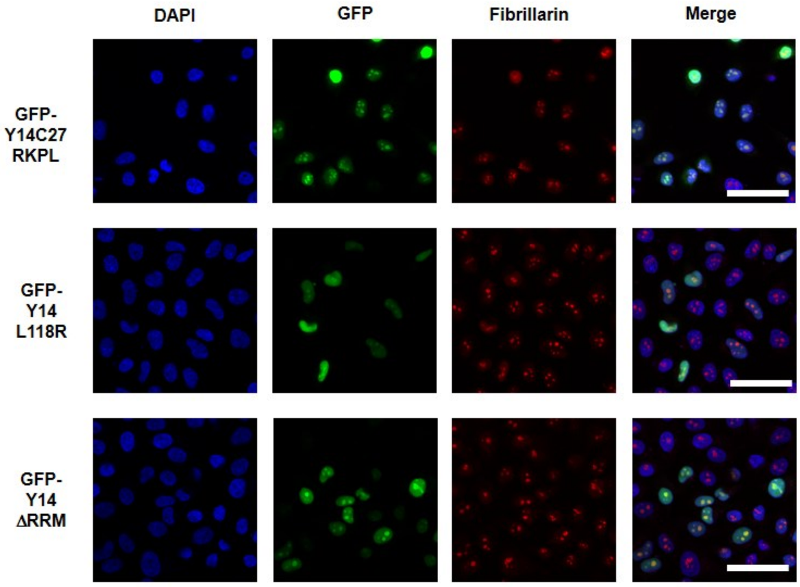
DAPI
GFP
Fibrillarin
Merge
GFP-
Y14C27
RKPL
GFP-
Y14
L118R
GFP-
Y14
ΔRRM

Fig. S4.

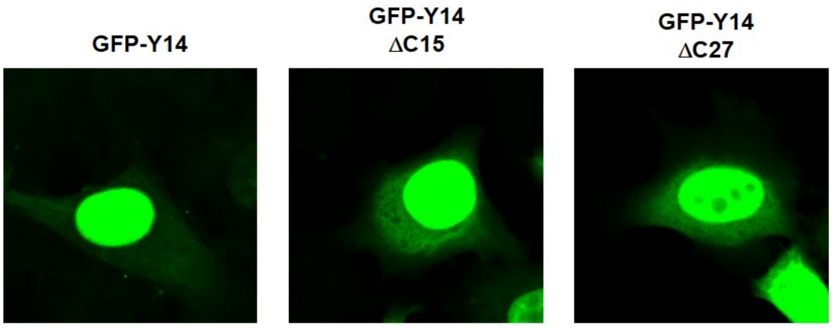

Fig. S5.

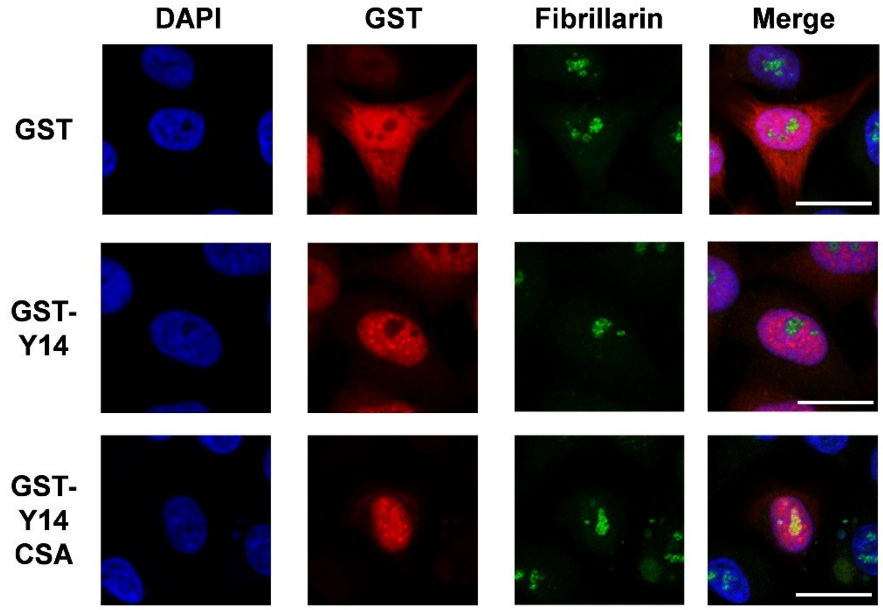

Fig. S6.

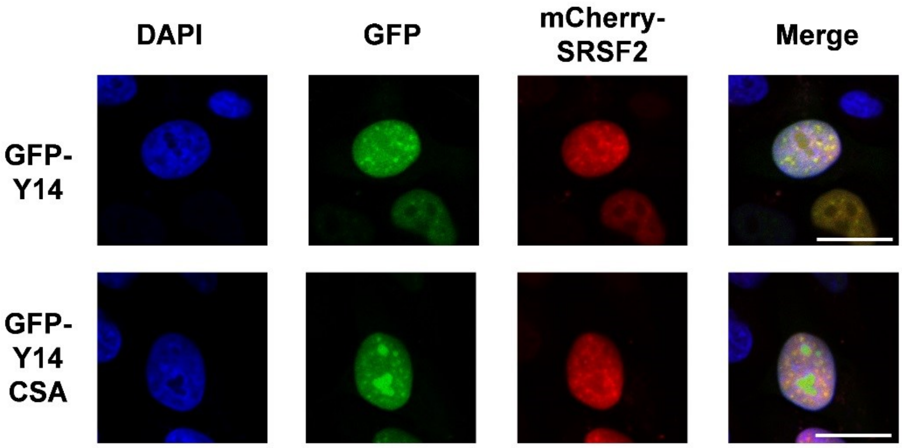

Fig. S7.

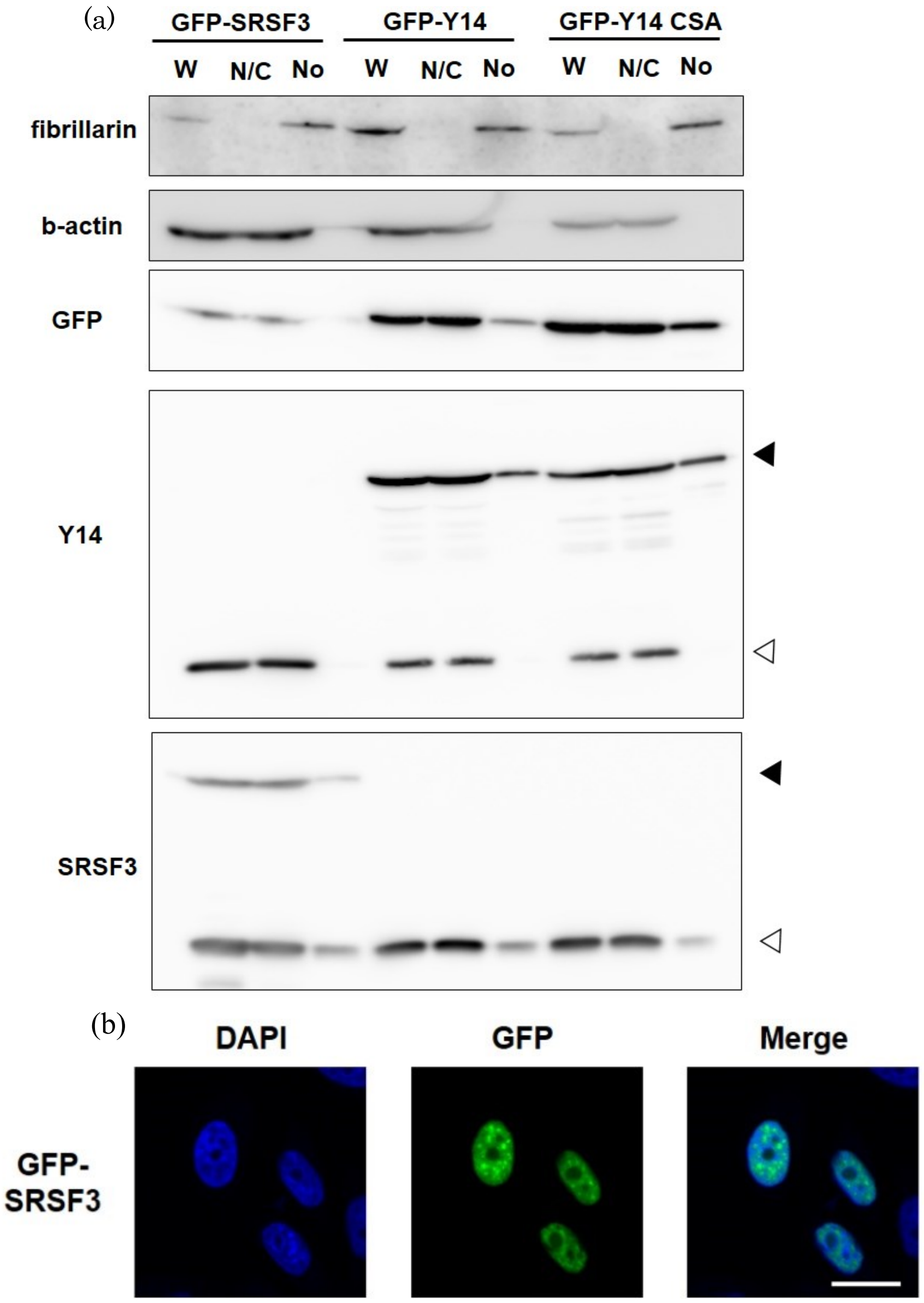
(a)
GFP-SRSF3
GFP-Y14
GFP-Y14 CSA
W
N/C
No
W
N/C
No
W
N/C
No
fibrillarin
b-actin
GFP
Y14
SRSF3
(b)
DAPI
GFP
Merge
GFP-SRSF3

Fig. S8.

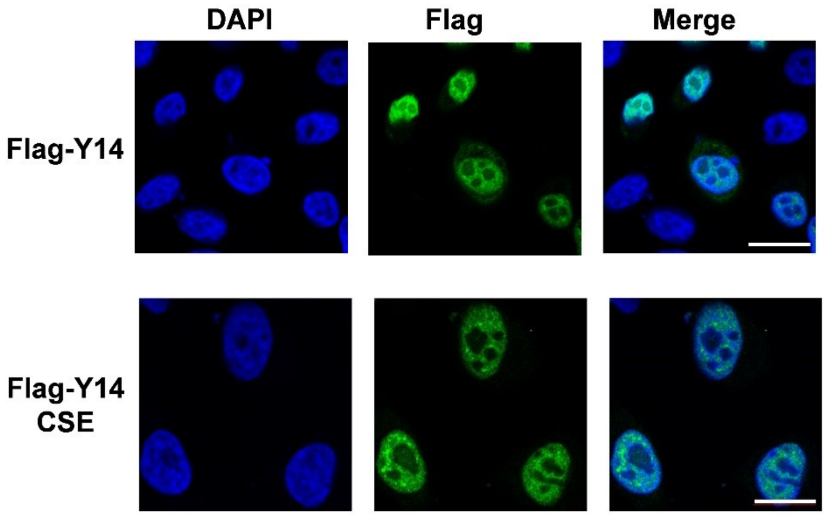

DAPI
Flag
Merge
Flag-Y14
Flag-Y14
CSE

Fig. S9.

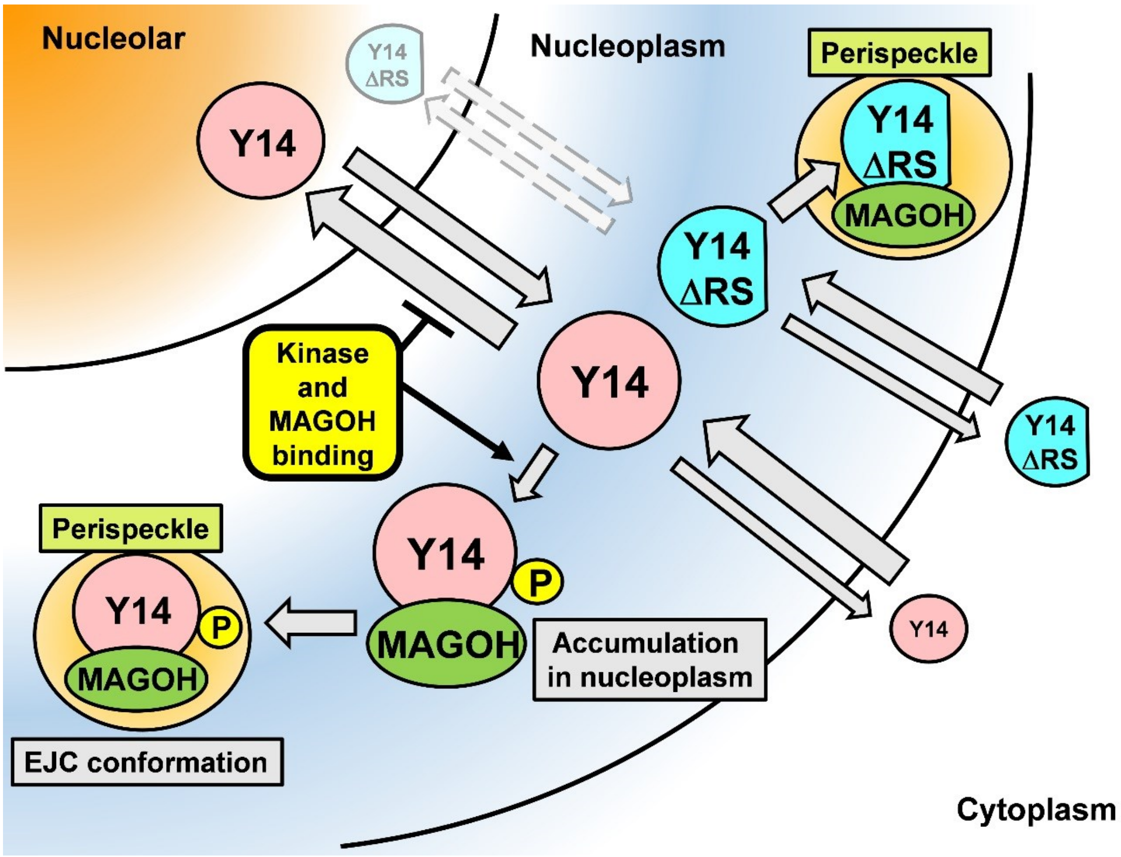

Nucleolar
Nucleoplasm
Perispeckle
Y14
ΔRS
MAGOH
Kinase
and
MAGOH
binding
Y14
P
Accumulation
in nucleoplasm
Perispeckle
EJC conformation
Cytoplasm

Fig. S10.

Original western blot of Fig. 1c

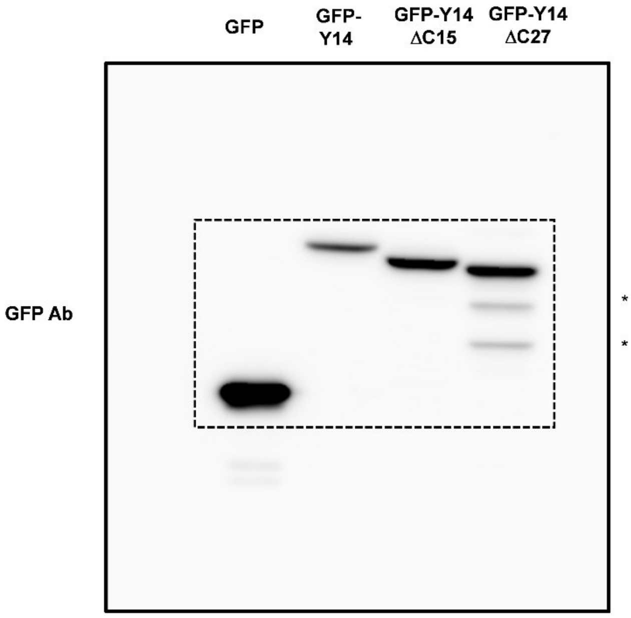

Original western blots of Fig. 2b

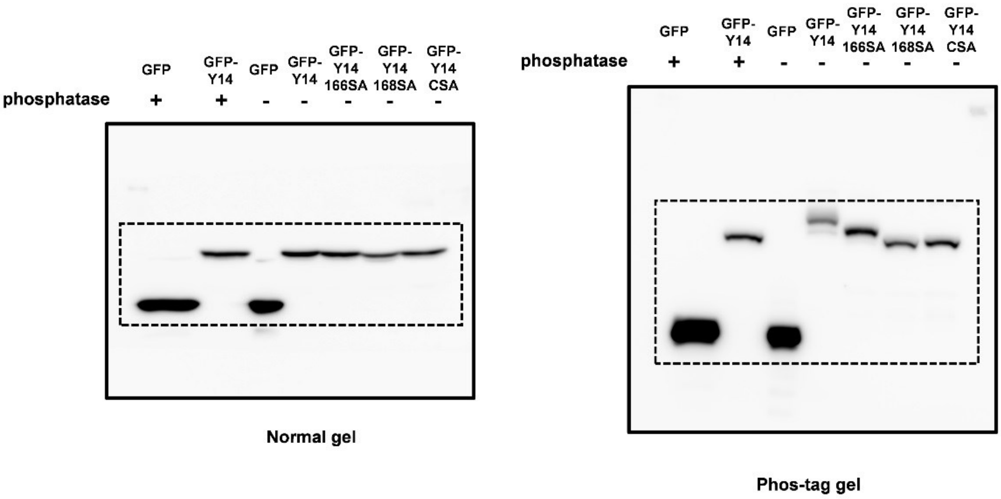

Original western blots of Fig. 2d

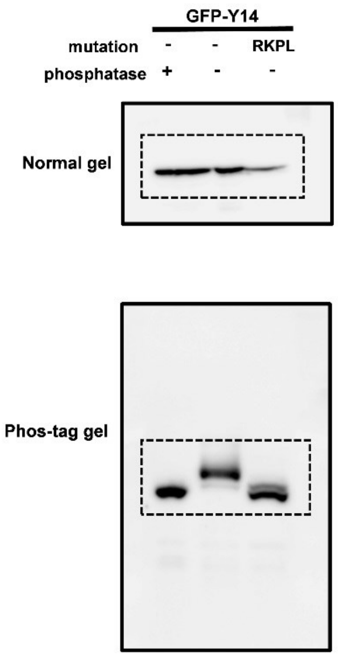

Original western blots of Fig. 3a

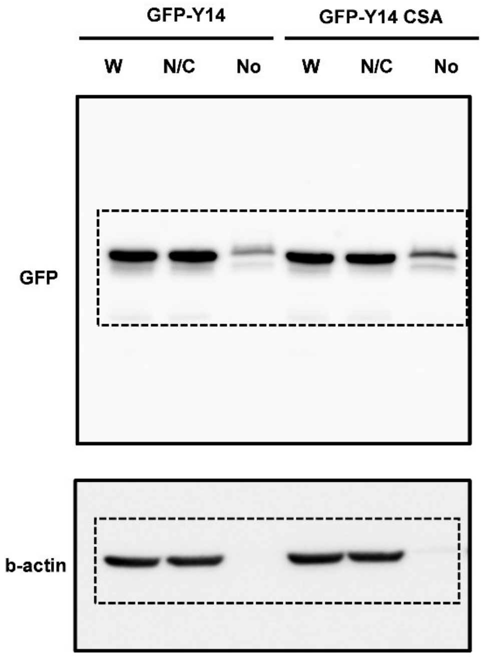

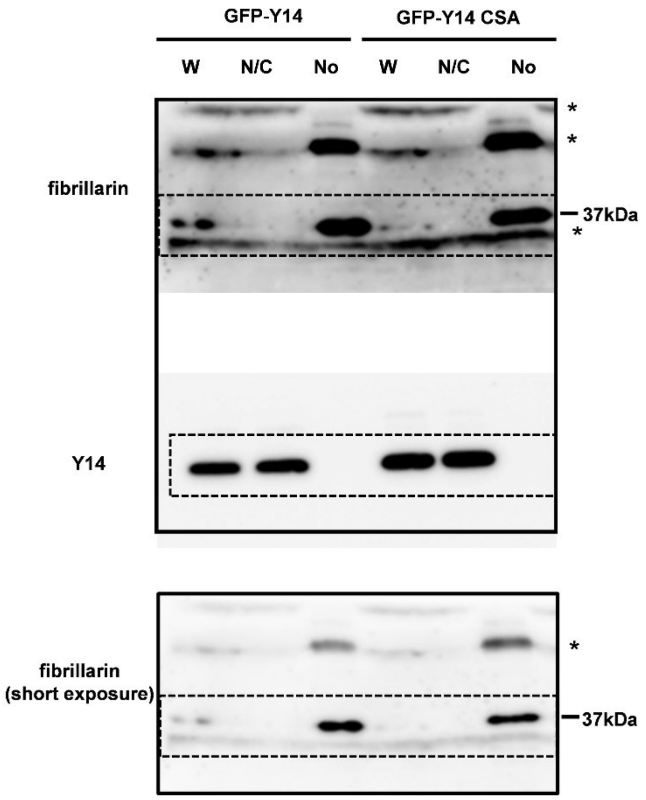

Original western blots of Fig. 4b

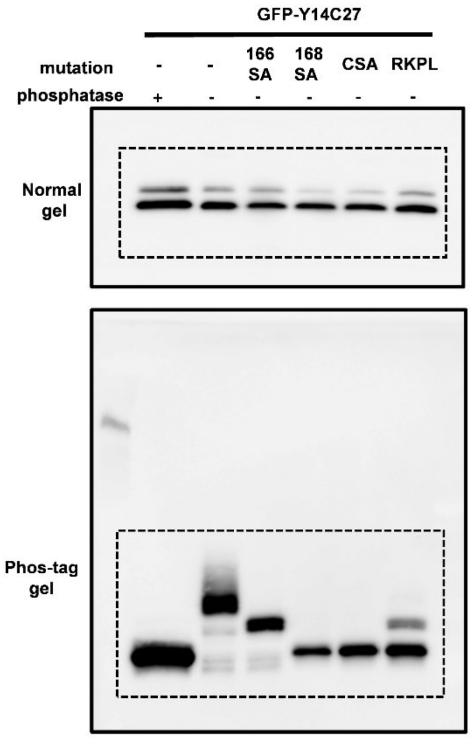

Original western blots of Fig. 5b and 5c

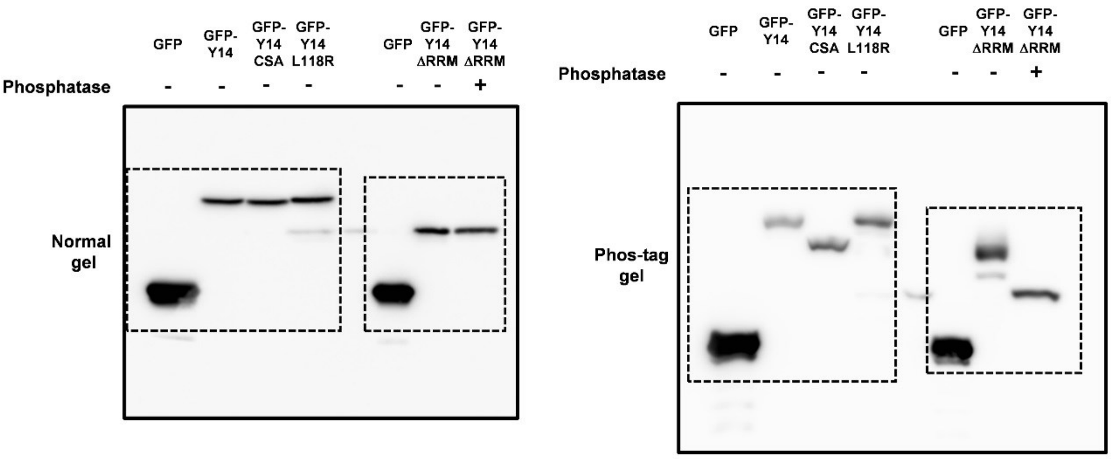

Reference

1. Fitzgerald, K. D. & Semler, B. L. Re-localization of cellular protein SRp20 during poliovirus infection: bridging a viral IRES to the host cell translation apparatus. PLoS Pathogens 7, e1002127 (2011).
